# Enhanced Electrical Integration of Engineered Human Myocardium via Intramyocardial versus Epicardial Delivery in Infarcted Rat Hearts

**DOI:** 10.1371/journal.pone.0131446

**Published:** 2015-07-10

**Authors:** Kaytlyn A. Gerbin, Xiulan Yang, Charles E. Murry, Kareen L. K. Coulombe

**Affiliations:** 1 Center for Cardiovascular Biology, University of Washington, Seattle, Washington, United States of America; 2 Institute for Stem Cell and Regenerative Medicine, University of Washington, Seattle, Washington, United States of America; 3 Department of Bioengineering, University of Washington, Seattle, Washington, United States of America; 4 Department of Pathology, University of Washington, Seattle, Washington, United States of America; 5 Department of Medicine/Cardiology, University of Washington, Seattle, Washington, United States of America; University of Toronto, CANADA

## Abstract

Cardiac tissue engineering is a promising approach to provide large-scale tissues for transplantation to regenerate the heart after ischemic injury, however, integration with the host myocardium will be required to achieve electromechanical benefits. To test the ability of engineered heart tissues to electrically integrate with the host, 10 million human embryonic stem cell (hESC)-derived cardiomyocytes were used to form either scaffold-free tissue patches implanted on the epicardium or micro-tissue particles (~1000 cells/particle) delivered by intramyocardial injection into the left ventricular wall of the ischemia/reperfusion injured athymic rat heart. Results were compared to intramyocardial injection of 10 million dispersed hESC-cardiomyocytes. Graft size was not significantly different between treatment groups and correlated inversely with infarct size. After implantation on the epicardial surface, hESC-cardiac tissue patches were electromechanically active, but they beat slowly and were not electrically coupled to the host at 4 weeks based on *ex vivo* fluorescent imaging of their graft-autonomous GCaMP3 calcium reporter. Histologically, scar tissue physically separated the patch graft and host myocardium. In contrast, following intramyocardial injection of micro-tissue particles and suspended cardiomyocytes, 100% of the grafts detected by fluorescent GCaMP3 imaging were electrically coupled to the host heart at spontaneous rate and could follow host pacing up to a maximum of 300–390 beats per minute (5–6.5 Hz). Gap junctions between intramyocardial graft and host tissue were identified histologically. The extensive coupling and rapid response rate of the human myocardial grafts after intramyocardial delivery suggest electrophysiological adaptation of hESC-derived cardiomyocytes to the rat heart’s pacemaking activity. These data support the use of the rat model for studying electromechanical integration of human cardiomyocytes, and they identify lack of electrical integration as a challenge to overcome in tissue engineered patches.

## Introduction

After a myocardial infarction, the death of cardiomyocytes results in compromised contractility of the heart, for which there is currently no cure. The development of cell-based regenerative therapies to replace human cardiomyocytes is a rapidly advancing area of research and includes the use of human pluripotent stem cells (hPSCs) and tissue engineering [1]. The leading pre-clinical strategy for transplantation of hPSC-derived cardiomyocytes is the use of dispersed cell suspensions delivered by needle injection into the left ventricular wall, which has been well-described in rodent models [2–5]. More recently the injection of dispersed cell suspensions has been used in larger animal models, and transplanted hPSC-cardiomyocytes have been shown to electrically couple to the host myocardium in the guinea pig (with a heart rate of 200–250 beats per minute [6, 7]) and the macaque monkey (with a heart rate of 80–120 beats per minute [8]). However, whether human PSC-derived cardiomyocytes can electrically couple to the rat heart is unknown, drawing into question the usefulness of this small animal model for studies of cardiac remuscularization.

Cardiac tissue engineering is a promising strategy to introduce a coherent mass of tissue onto the heart for muscular regeneration, and it provides the ability to engineer the micro- and macroscopic architecture of the tissue [9–13]. Scaffold-based engineered tissues have been shown to align cardiomyocytes to promote anisotropic electrical conduction and improve contractile function [13, 14], and scaffold-free approaches such as cell sheets and our described self-assembly methods recapitulate many physiological functions with endogenous cells creating the extracellular matrix environment [15, 16]. Engineered cardiac tissues are typically attached onto the epicardial surface of the heart with sutures or an adhesive [16–22]. In our experience, however, engineered heart tissue patches placed on the epicardium are often separated from the host myocardium by scar tissue, raising questions about their ability to form gap junctions with host myocardium that are required for electrical integration. Additionally, the need for surgical placement of patches reduces the number of clinical patients who could potentially be treated compared to a minimally-invasive, catheter-based delivery approach.

In this study we sought to address the potential limitations of epicardial placement of engineered tissue while retaining the advantages that tissue engineering offers, such as control over microscale architecture and lack of enzymatic dispersion of cells prior to implantation. We developed scaffold-free, engineered cardiac “micro-tissue particles” by self-assembly of human embryonic stem cell (hESC)-derived cardiomyocytes in microwells. These micro-tissue particles have a well-defined micron scale spherical diameter (<200 μm) and can be delivered via needle injection into the injured myocardial wall. In this study, three different delivery strategies (dispersed cell cardiomyocyte injection, micro-tissue particle injection, and engineered cardiac tissue patch implantation) were assessed for engraftment and electrical integration with the injured rat myocardium. No other studies directly compare graft integration between diverse delivery strategies such as here, where dispersed cells are used as a positive control for engraftment and engineered tissues are delivered either intramyocardially or onto the epicardium. While all approaches yielded comparable graft sizes, the epicardial patches did not integrate electrically with the host myocardium as detected via fluorescence imaging of the cell-autonomous, genetically encoded calcium indicator protein GCaMP3. In contrast, following intramyocardial delivery, both micro-tissue particles and dispersed cell grafts coupled electrically with the rat heart and could be paced through the host tissue up to 6.5 Hz. This suggests that electrophysiological adaptation of hESC-derived cardiomyocytes occurs *in vivo* and supports the use of the rat ischemia/reperfusion model for cardiac remuscularization studies using hPSC-derived cardiomyocytes.

## Materials and Methods

### Ethics Statement

All animal procedures were conducted in accordance with the US NIH Policy on Humane Care and Use of Laboratory Animals and the UW Institutional Animal Care and Use Committee (IACUC), who approved this study (protocol #2225–04). A surgical plane of anesthesia was maintained by IP ketamine/xylazine for myocardial infarction or inhaled isoflurane for hESC-cardiomyocyte implantation. Buprenorphine was used for post-operative analgesia. Overdose of pentobarbital/phenytoin solution was used for euthanasia.

### Human Embryonic Stem Cell-Derived Cardiomyocyte Culture and Differentiation

All cardiomyocytes in this study were derived using H7 hESCs (WA07, WiCell Research Institute, Madison, WI) or RUES2 cells (The Rockefeller University, New York, NY), which were genetically engineered to express GCaMP3 as described elsewhere [6, 8]. Undifferentiated GCaMP3 hESCs were maintained in culture in feeder-free conditions on Matrigel in mouse embryonic fibroblast (MEF)-conditioned media supplemented with 5 ng/ml basic fibroblast growth factor (bFGF). Cardiomyocyte differentiation was induced using an established protocol [2] in a high-density cell monolayer with addition of activin A and BMP4 in RPMI 1640 basal medium (Invitrogen) with B27 Supplement minus insulin (Invitrogen) with minor modifications: the small molecule GSK3-inhibitor CHIR99021 (Cayman Chemicals) was added at 1 μM one day prior to activin A (R&D Systems; 100 ng/mL) with 1x Matrigel (BD Biosciences) and at day 1 (1 μM) with BMP4 (R&D Systems; 5 ng/mL) for 48 hours. The Wnt inhibitor XAV939 (Tocris) was added at day 3 for 48 hours. Fluorescence activated cell sorting (FACS) was used to characterize the differentiated cell population. Briefly, cells were fixed with 4% paraformaldehyde and incubated with a cardiac troponin T (cTnT) antibody, followed by incubation with a PE-conjugated secondary antibody. Fluorescence characterization was performed on a BD FACS Canto II (BD Biosciences) and subsequently analyzed using FloJo software. Only cultures having >50% cTnT-positive cells were used for *in vivo* implantation.

### Cryopreservation and Thawing of hESC-Cardiomyocytes

Cardiomyocytes used in this study were cryopreserved on day 21–24 of differentiation and thawed immediately prior to engineered tissue formation or cell injection, following a previously described protocol [23]. One day prior to cryopreservation, cells were heat shocked for 1 hour at 42°C. Prior to enzymatic dispersion, the ROCK inhibitor Y-27632 (10 μM) was added to the culture medium for 1 hour, and cells were dispersed by incubation with 0.25% trypsin in EDTA. Cardiomyocytes were resuspended in CryoStor (Stem Cell Technologies) and frozen in cryovials in a controlled rate freezer to -80°C before being stored in liquid nitrogen. To thaw cryopreserved cells, cryovials were thawed briefly at 37°C followed by addition of RPMI+B27+insulin with 200 U/ml DNAse. Cells were washed and resuspended in either RPMI+B27+insulin with Y-27632 (10 μM) for micro-tissue particle or patch formation, or in an RPMI-based pro-survival cocktail [2] for cell implantation containing 50% (vol/vol) growth factor-reduced Matrigel, 100 μM ZVAD (benzyloxycarbonyl-Val-Ala-Asp(O-methyl)-fluoromethyl ketone, Calbiochem), 50 nM Bcl-X_L_ BH4 (cell-permeant TAT peptide, Calbiochem), 200 nM cyclosporine A (Novaritis), 100 ng/mL IGF-1 (Peprotech), and 50 μM pinacidil (Sigma).

### Micro-Tissue Particle and Cardiac Patch Formation

Immediately after cell thawing, cardiac micro-tissue particles were formed in polydimethylsiloxane (PDMS) microwells using AggreWell 400 plates (Stemcell Technologies). Ten million hESC-cardiomyocytes were distributed by centrifugation into 8 wells containing approximately 1,200 microwells each, yielding approximately 1000 cells/micro-tissue particle. Micro-tissue particles formed overnight and were used for implantation one day after plating. For cardiac patch formation, 10 million cardiomyocytes were seeded into custom made round-bottom PDMS molds in 6-well plates and allowed to settle for 1 hour at 37°C before additional RPMI+B27+insulin was added, incubated overnight, and then maintained in rotating suspension culture for 6–7 days prior to implantation with medium changes every other day. Micro-tissue particles and cardiac patches were heat shocked for 1 hour at 42°C the day before implantation and incubated in 200 nM cyclosporine A and 100 ng/mL IGF-1 overnight. For implantation, engineered tissues were harvested by gently pipetting media to wash particles and patches out of the PDMS molds as necessary, suspended in pro-survival cocktail as described above, and kept on ice until implantation.

### Ischemia/Reperfusion Injury and Cardiomyocyte Implantation

All animal procedures were conducted in accordance with the US NIH Policy on Humane Care and Use of Laboratory Animals and the UW Institutional Animal Care and Use Committee (IACUC). Two experimental groups (patch and micro-tissue particle) and two control groups (cell injection and sham) comprised the study design. Eight rats were enrolled per engraftment group and three rats enrolled in the sham group. Eight-week old (250g) male athymic Sprague-Dawley rats (Charles River) were anesthetized with intraperitoneal injection of 90 mg/kg ketamine and 6 mg/kg xylazine, intubated, and mechanically ventilated. The rat was placed on an electric warming pad with temperature feedback control via a rectal probe to maintain core body temperature at 37°C. A thoracotomy exposed the heart, and the left anterior descending (LAD) coronary artery was occluded for 60 minutes, reperfused, and the chest was aseptically closed. For implantation surgeries, animals were randomized and no exclusion criteria were used. Four days after ischemia/reperfusion injury, rats were anesthetized with up to 5% isoflurane supplemented with oxygen, intubated, and mechanically ventilated. A second thoracotomy was used to open the chest and dispersed single cells or micro-tissue particles suspended in 90 μL of pro-survival cocktail were injected into the center of the infarcted left ventricle wall and each lateral infarct border zone (10x10^6^ cells total input cells, 3 injections, 30 μL each, purse-string suture with 8–0 suture). Sham control rats received intramyocardial injection of vehicle only. A 29-gauge needle was used for dispersed-cell and sham injections, and a 24-gauge needle was used for micro-tissue particle injections. For epicardial patch implantation, the patch was bathed in pro-survival cocktail for 1 hour prior to being placed over the infarct. A single 8–0 suture was passed through the myocardium and the hESC-cardiac tissue patch and held in place for 1 minute prior to tie-off to allow for attachment (likely via blood clot), followed by up to 3 additional sutures being placed. Some patches were broken during the implantation procedure due to difficulty handling the compliant tissue, resulting in a fragmented patch or multiple pieces of the patch being sutured onto the heart’s surface. This is a potential source of cell loss and will require future improvements in procedure. The chest was closed and animal recovery was monitored. Rats received analgesic (buprenorphine) for 2 days post-MI and post-implant surgery. All animals received a subcutaneous injection of 0.75 mg cyclosporine A for 7 days beginning the day before implantation, as per the established pro-survival cocktail protocol [2].

### Ex Vivo Fluorescent Imaging of GCaMP3 Micro-Tissue Particle Grafts

Hearts were collected 4 weeks after hESC-cardiomyocyte implantation and mounted on a Langendorff apparatus perfused with modified Tyrode solution at 37°C as described previously [6, 8]. 2,3-butanedione monoxime (BDM, 12–20 mM) was used to mechanically arrest the heart, and the GCaMP3 fluorescent signal was visualized using an EXFO X-Cite illumination system mounted on an epifluorescent stereomicroscope (Nikon, SMZ 1000). The fluorescent signal was captured and recorded by a CCD camera (Andor iXon 860 EM-CCD, Andor Solis software) along with the heart ECG (recorded in LabChart). In some cases, multiple graft regions were identified within the same rat heart. The heart was considered to be “coupled” when each region demonstrated 1:1 coupling between the fluorescent signal and rat ECG at spontaneous rates. These grafts were subsequently challenged with electrical pacing to determine the maximum capture rate (MCR) of the graft. The fastest MCR from any region within a single heart was taken as the MCR for the heart. Grafts were electrically paced through the host myocardium via insertion of electrodes, and data analysis was performed using Andor software and LabChart as described elsewhere [6]. At the end of the imaging experiment, permanent medical marking dye (Bradley Products, Inc.) that survives histological processing was used to mark the location of imaged grafts.

### In Vitro Electrical Stimulation of hESC-cardiomyocytes

HESC-cardiomyocytes at 21–24 days of differentiation were replated in triplicate into 6-well plates coated with Matrigel for field stimulation at 1 or 6 Hz (5 V/cm, 4 ms pulse width) using the C-Pace Culture Stimulator (IonOptix) and compared to unstimulated control hESC-cardiomyocytes. Measurements of spontaneous rate, excitation threshold (V), and maximum capture rate (Hz) were made at 2, 4, and 6 weeks of stimulation. Responses to stimulation were observed in 2–3 wells for all conditions except the unstimulated control hESC-cardiomyocytes at the 4-week time point, where only 1 of 3 wells was beating and responding to stimulation.

### Immunohistochemical Analysis

Histological stains and subsequent analysis were conducted as described previously by our group [2, 8]. Briefly, hearts were perfused with 150 mM KCl solution after *ex vivo* imaging, fixed overnight in 4% paraformeldehyde, sliced into 2 mm-thick sections, and then processed, sectioned, and stained with the appropriate primary and secondary antibodies. Infarcts were visualized by staining with picrosirius red and fast green counterstain and quantified by measuring the area of collagenous scar labeled by picrosirius red within each section, normalized to left ventricular area. To quantify graft size, sections were incubated overnight with rabbit anti-GFP antibody (Novus) followed by a one-hour incubation with Alexa Fluor-488 goat anti-rabbit (Molecular Probes) for immunofluorescent imaging or with an avidin-biotin goat anti-rabbit antibody reaction and developed with diaminobenzadene (DAB, Vector Labs) for bright-field images. To ensure that all engraftment areas were identified by histology, four sectioning planes were analyzed for each 2 mm-thick slice of the heart, where each sectioning plane was 150–200 μm apart. Cardiac purity and further analysis of engrafted cells was assessed on sections double-stained for GFP and alpha-actinin (mouse monoclonal, Sigma-Aldrich) or connexin 43 (rabbit polyclonal, Sigma) with corresponding Alexa Fluor secondary antibodies (Molecular Probes).

### Statistical Analysis

All immunohistochemical sections were imaged and measurements were made using ImageJ. Scar size was identified by picrosirius red staining and measured in the 3 most apical sections, spaced 2 mm apart, and reported normalized to left ventricular area (percent). Graft size was determined in five histological sections, spaced 2 mm apart from apex to base of hearts, and all 8 animals in each treatment group were included in the analysis, including one animal per group that showed zero graft regions in an “intent-to-treat” analysis. Anterior wall thickness was measured across the picrosirius red-positive infarct region in 3 locations per single heart section at the third sectioning plane and averaged for each heart. Patch thickness was not included in these measurements. Average MCR within each group is reported for each heart where a graft was detected by GCaMP imaging. Statistical significance (P<0.05) was determined by one-way ANOVA where significant differences followed by a two-tailed Student’s t test assuming unequal variance and for calculations of Pearson’s correlation coefficient in Graphpad Prisim software. All values are reported as means, and error bars represent SEM.

## Results

### Formation of Engineered Cardiac Tissues

**Fig 1:**
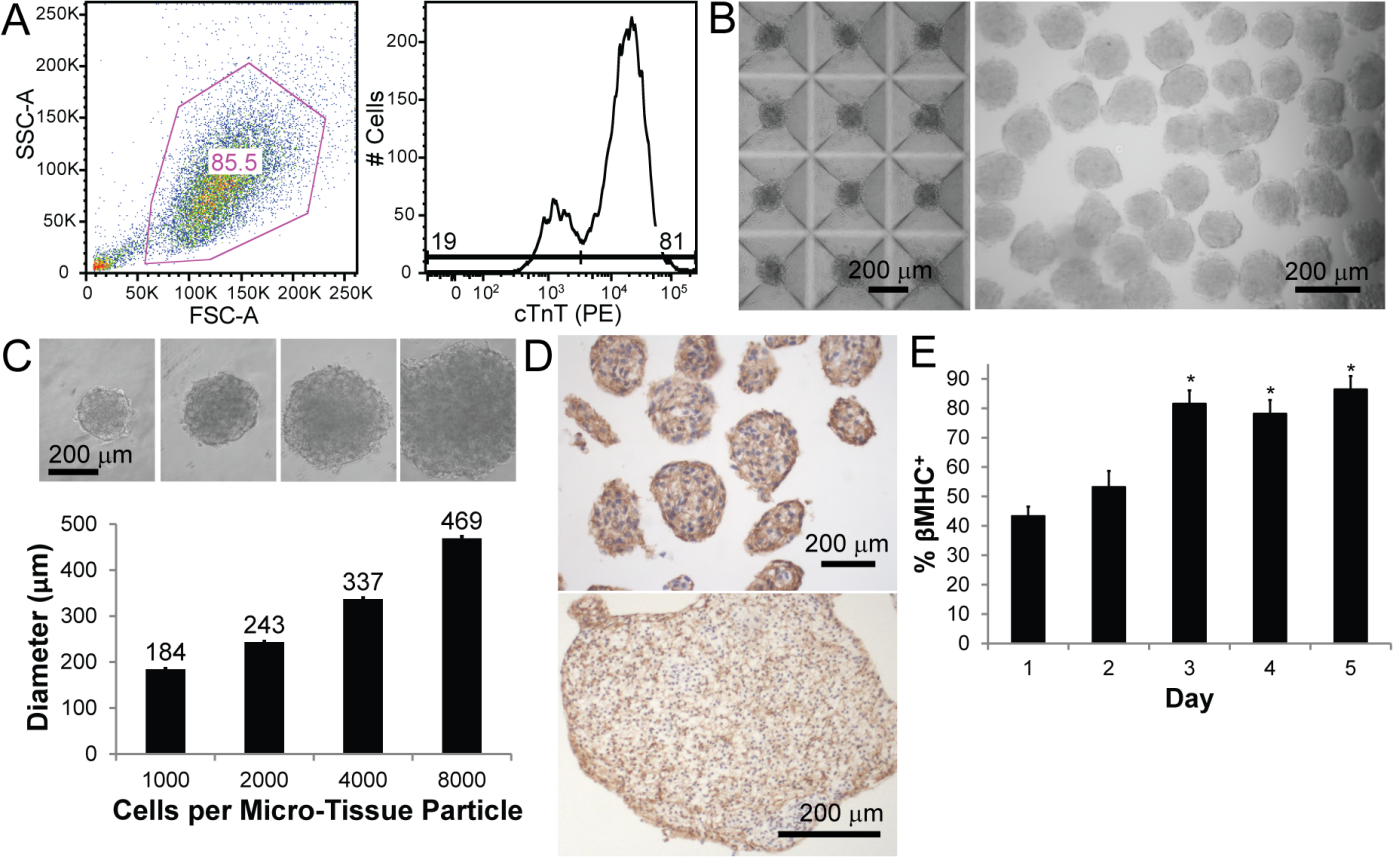
Formation of hESC-derived cardiac engineered tissues. (A) hESCs are differentiated into cardiomyocytes with high efficiency as characterized by flow cytometry analysis. An example cell population (85.5% single cell population, left) shows 81% expression of cardiac troponin T (cTnT, PE fluorescence, right) relative to isotype control (not shown). (B) Micro-tissue particles (MTPs) are formed by seeding approximately 1000 cells per microwell (left) and are easily removed from molds by a gentle media wash (right). (C) *In vitro* characterization of MTPs indicates highly defined particle diameter based on cell input number. (D) Cardiac engineered tissues have high cardiac purity as indicated by β-myosin heavy chain (β-MHC; brown, DAB) in MTPs (top) and cardiac patches (bottom). (E) Cardiac purity by β-MHC staining shows increasing purity with culture time. * P < 0.05 versus Day 1.

Human cardiomyocytes derived from hESCs were formed into scaffold-free injectable micro-tissue particles or macroscopic patches and characterized *in vitro* prior to implantation. The input cardiomyocyte purity was assessed by flow cytometry for the definitive cardiac marker cardiac troponin T (cTnT) and showed 50–94% pure populations (average 70.2 ± 14.2%; Fig 1A), and monolayer cultures exhibited spontaneous contraction and showed a robust GCaMP3 fluorescence with each contractile cycle (S1 Video). Micro-tissue particles formed overnight and showed uniform diameter based on input number of cells per particle (Fig 1B and 1C). For intramyocardial injection we chose a micro-tissue particle diameter of <200 μm, corresponding to 1000 cells per particle, in order to minimize risk of shear stress damage to cardiomyocytes when implanted using a 24 gauge needle (inner diameter of 311 μm). Engineered tissues exhibited robust contraction and GCaMP3 fluorescence (S2 Video and S3 Video), and histological staining for β-myosin heavy chain (β-MHC) demonstrates high cardiac purity of the population of micro-tissue particles and patches used for implantation (Fig 1D). As was previously observed for cardiac patches [24], culturing micro-tissue particles in RPMI+B27+insulin medium causes increased cardiac purity over time in culture (Fig 1E), although only 1-day old micro-tissue particles were used for injection *in vivo*.

### Engraftment of hESC-Cardiomyocytes in Ischemia/Reperfusion-Injured Hearts

**Fig 2:**
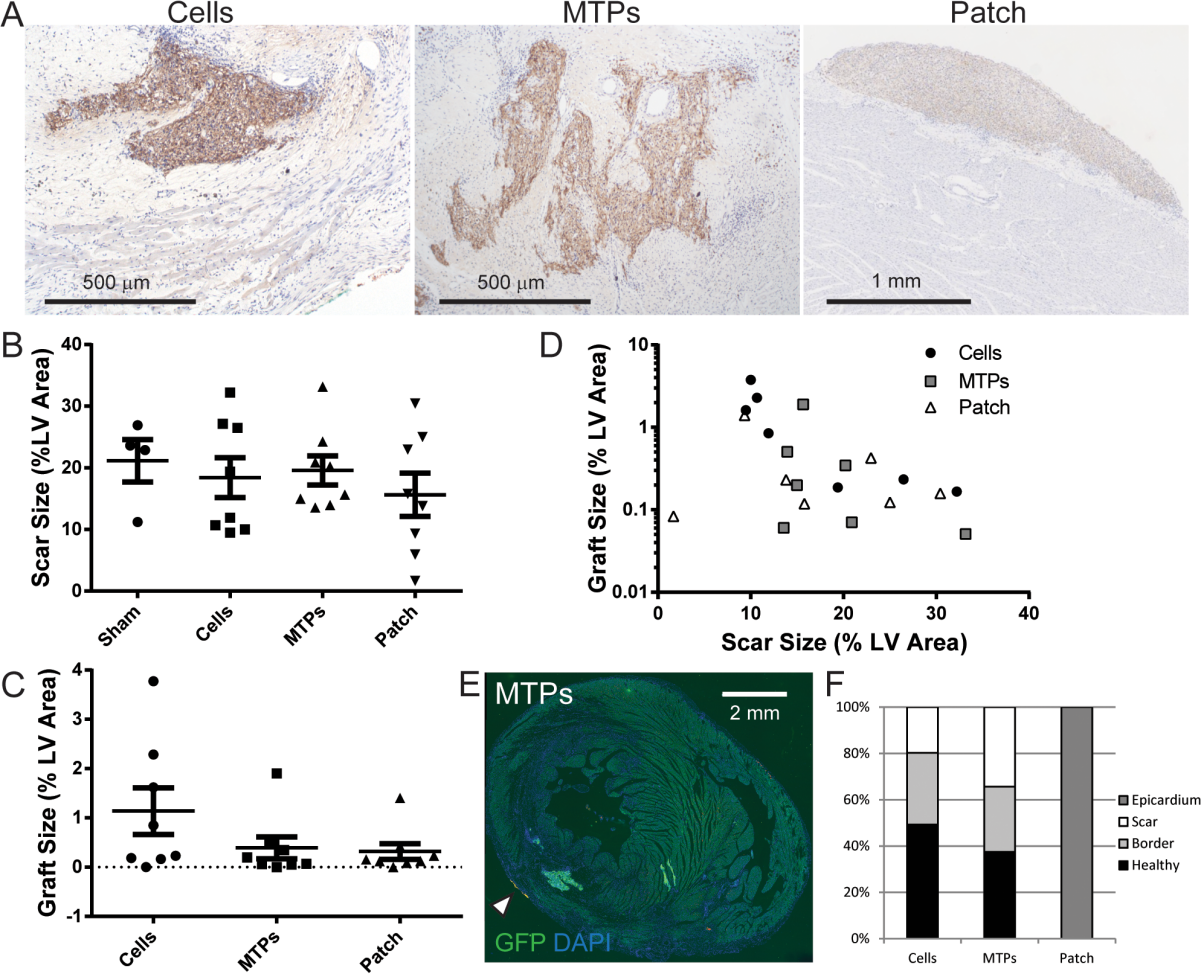
Cardiomyocyte engraftment in injured rat hearts at 4 weeks. (A) Human cardiac grafts are identified by immunohistochemistry for GFP (brown, DAB) that binds to the GCaMP3 protein for the cell, micro-tissue particle (MTP), and patch groups. Hematoxalyin nuclear counterstain (blue). (B) Infarct scar area quantified by picrosirius red-positive area shows no difference between groups, normalized to left ventricular (LV) area at 4 weeks. (C) Graft size measured by GFP-positive graft area is normalized to LV area and is equivalent between groups (n = 8/group). (D) GFP-positive graft size declines with larger scar size for the cell grafts identified by histological analysis using Pearson correlation analysis (see text), but is weak and not significant in the micro-tissue particle or patch groups. (E) Micro-tissue particles (GFP, green) engrafted in the infarct region and lateral border within the septum of a rat heart are shown at 4 weeks stained for GFP (green) with DAPI (blue) nuclear stain. Topically applied dye (yellow-orange) marks the location of where an intramyocardial graft was detected by *ex vivo* imaging (arrow head). (F) Analysis of graft distribution in the heart indicates that cells and micro-tissue particles engraft in the scar, border zone, and healthy myocardium with equal distribution, while patch implants are found on the epicardium.

**Fig 3:**
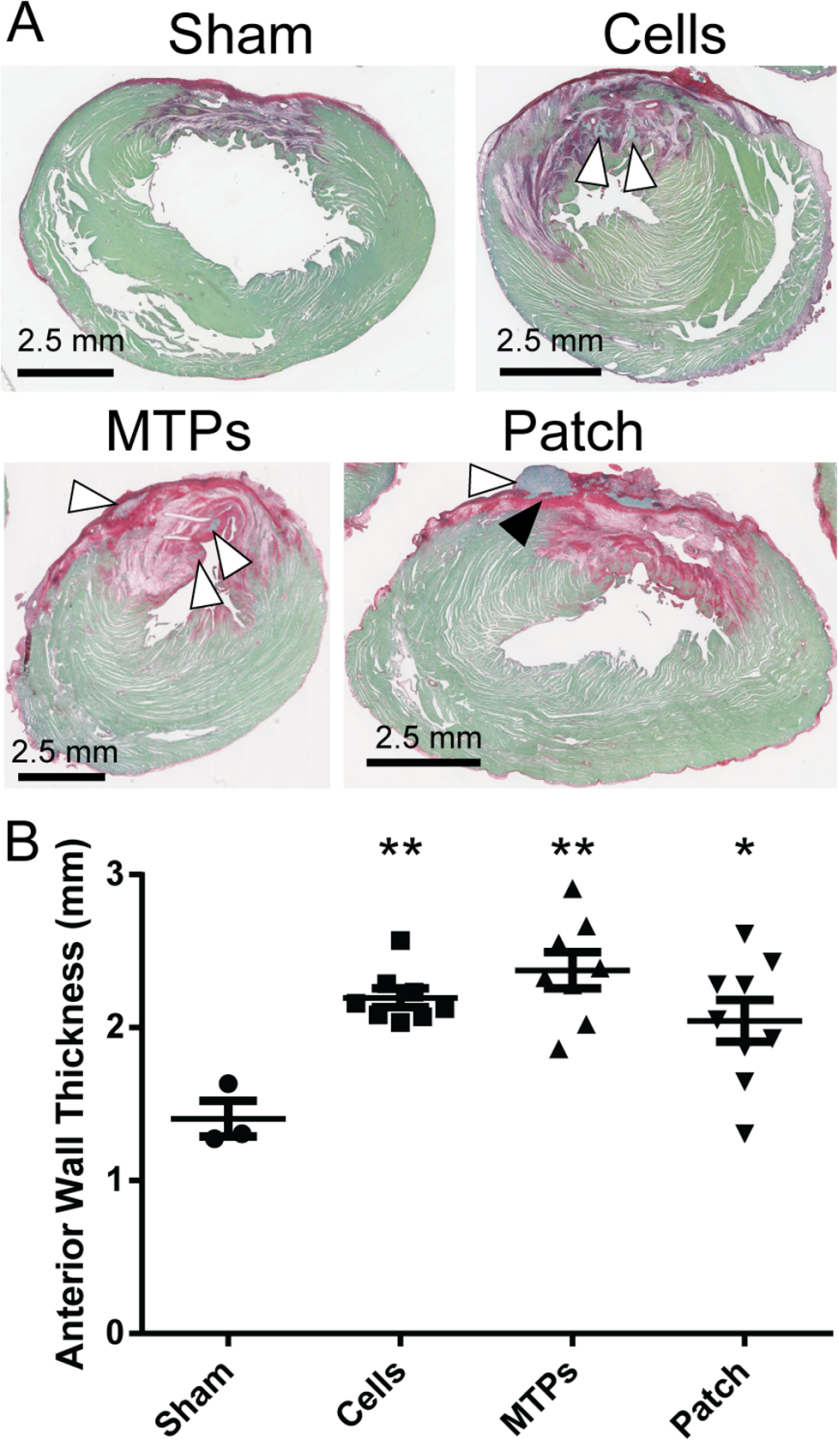
Engrafted cardiomyocytes improve infarct wall thickness. (A) Representative image of infarcts by picrosirius red (collagen) with fast green counterstain are shown for sham control, cell injection, micro-tissue particle injection, and patch implants. White arrow heads identify grafts, and black arrow head identifies picrosirius red-positive scar region that separates patch graft from healthy host myocardium. (B) Anterior wall thickness measured by histology is preserved in all three treatment groups compared to sham control. Thickness of patch implants was not included in anterior wall thickness measurements. * P < 0.05; ** P < 0.01.

**Fig 4:**
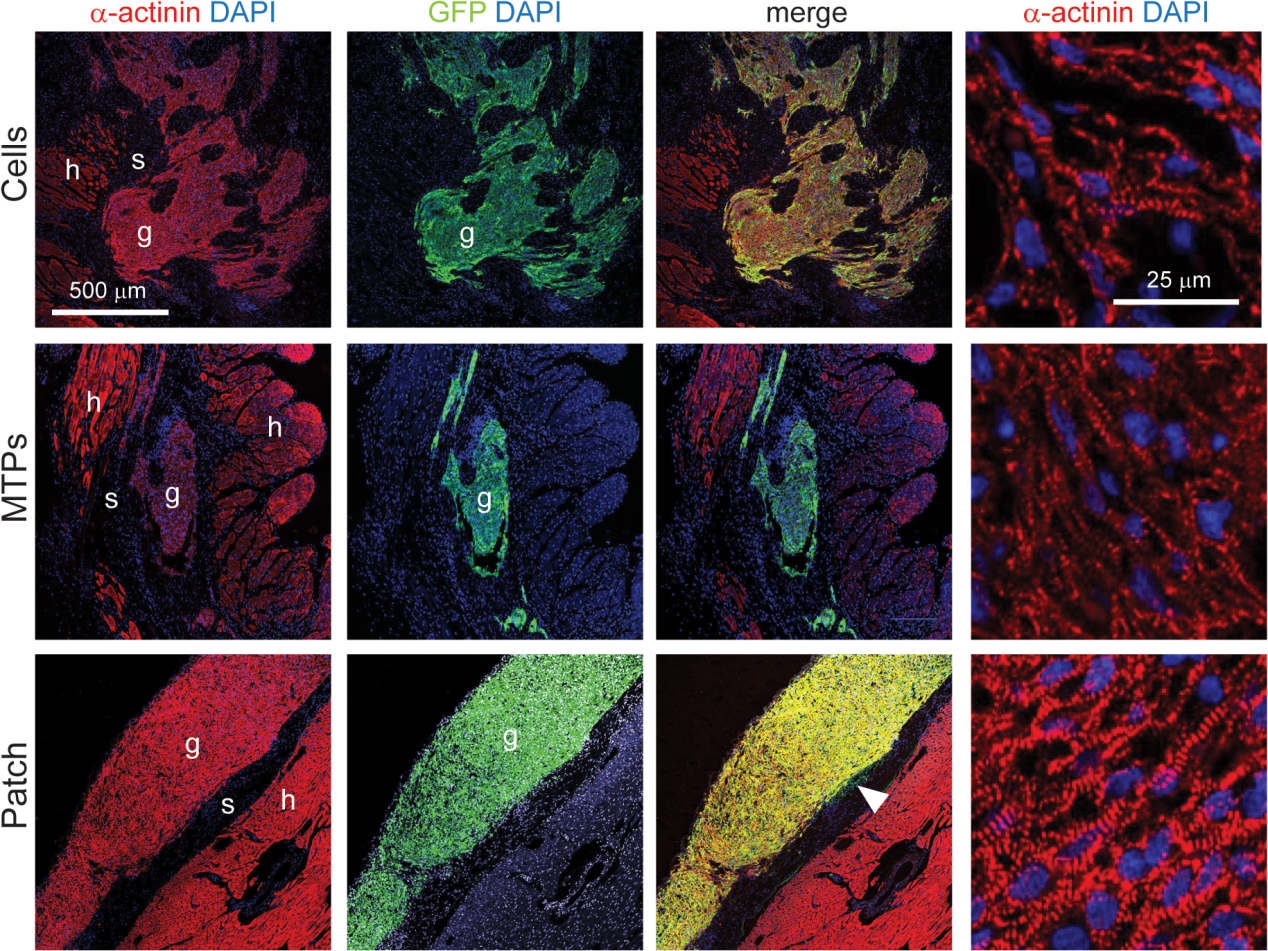
Human grafts contain striated cardiomyocytes. Confocal fluorescent imaging of engrafted hESC-cardiomyocytes at 4 weeks indicate that all three engraftment methods produce grafts that have high cardiac purity, as indicated by a double-positive stain for α-actinin (red) and GFP (green) with nuclear DAPI (blue), in representative images of all grafts. All grafts demonstrate sarcomere striations at higher magnification (far right). Scar tissue appears as the dark band separating the patch graft and host myocardium (see Fig 3 for picrosirius red labeled scar, black arrowhead) and this extends to the edges of the patch (outside the field of view; not shown). White arrowhead in patch image points to GFP^+^/α-actinin^-^ non-cardiac cells. g, graft; s, scar, h, host. Scale bar = 500 μm and 50 μm (far right column).

Immunostaining for GFP was used to detect the GCaMP3 transduced hESC-cardiomyocytes four weeks after transplantation. In each of the three implantation groups, engrafted hESC-cardiomyocytes were identified in 7 of 8 hearts (87.5%). Morphological assessment of grafts shows dense, highly cardiac GFP-positive graft regions within the ventricular wall for dispersed cells and micro-tissue particles and on the epicardial surface for cardiac patches (Fig 2A). Infarct size was not different between sham operated animals and any of the treatment groups (P = 0.70) by picrosirius red staining of the collagenous scar (Fig 2B, Table 1, and Fig 3A), as previously observed for hESC-cardiomyocyte injections versus negative controls [2]. We had hypothesized that the engineered tissue constructs would yield larger grafts compared to dispersed cell grafts. Surprisingly, despite a thorough histological analysis, we found that graft size was not statistically different between the three implantation groups (Fig 2C and Table 1). This may in part reflect fragmentation of some patches during implantation, and if so, the development of more robust surgical implantation techniques may improve graft size, as reported by other groups [17]. There was a moderate, negative correlation between graft size and infarct size for cell grafts (r = −0.76, P = 0.03, R^2^ = 0.58), which was also observed globally when all three treatment groups were analyzed together (r = −0.41, P<0.05, R^2^ = 0.17). Although the MTP and patch groups show approximately the same relationship, the correlation was weak and did not reach significance for MTP (r = −0.36, P = 0.39, R^2^ = 0.13) or patch grafts (r = −0.12, P = 0.78, R^2^ = 0.01) likely due to small sample size or possibly due to treatment modality (Fig 2D). Larger infarcts appear to be inhospitable to grafts, perhaps due to graft ischemia or deleterious paracrine signals. The intramyocardial implants are found throughout the myocardial wall (Fig 2E), including in the scar region. Distribution of the intramyocardial grafts between the scar, border zone, and healthy tissue was not different for the dispersed cardiomyocyte and micro-tissue particle injection groups (Fig 2F). Anterior wall thickness of the left ventricle was increased in all implantation groups compared to sham operated control despite the persistence of scar that often separated grafts from healthy myocardium (Fig 3). Histological analysis of the grafts by co-localization of GFP (from the GCaMP3 molecule) and α-actinin demonstrated that the majority of engrafted cells were cardiomyocytes with visible sarcomere striations, and with rare yet detectable areas of surviving non-cardiac cells (arrowhead in patch image) for all three implantation groups (Fig 4). Cardiac patches were the only grafts that maintained most of their initial shape to produce a uniform, thick mass of transplanted hESC-cardiomyocytes, as opposed to both dispersed cell and micro-tissue particle injections that show disjointed graft areas within the myocardial wall.

**Table 1:** Summary of hESC-cardiomyocyte engraftment at 4 weeks by histology and *ex vivo* fluorescent imaging. No significant differences exist between treatment groups. MCR, maximum capture rate; NA, not applicable.

Group	Scar Size (% LV area)	Graft Size (% LV Area)	Graft Size (% Scar Area)	Grafts Detected by Ex Vivo Imaging	Detected Grafts Coupled to Host	MCR Range (Hz)	Average MCR (Hz)
**Dispersed Cells**	18.4 ± 3.2	1.1 ± 0.5	9.7 ± 4.5	4/8 hearts	4/4 grafts	5.5–6.5	6.1±0.2
**Micro-Tissue Particles**	19.6 ± 2.4	0.4 ± 0.2	2.5 ± 1.2	6/8 hearts	6/6 grafts	5.0–6.0	5.4±0.2
**Patches**	15.6 ± 3.5	0.3 ± 0.2	10.8 ± 9.5	6/8 hearts	0/6 grafts	NA	NA

### Electrical Coupling of Graft to Host

**Fig 5:**
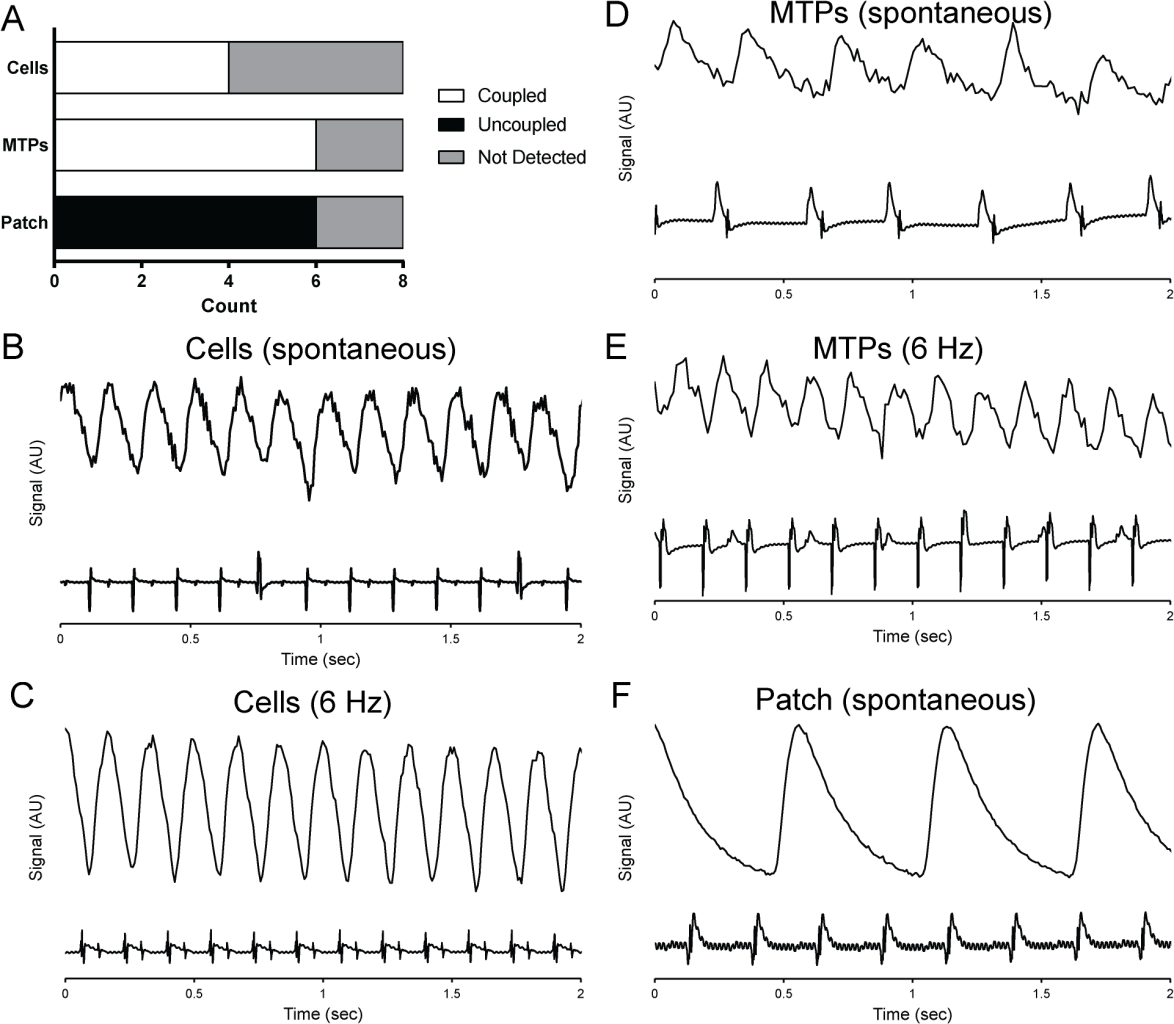
Assessment of human myocardial graft coupling to host heart by GCaMP3 *ex vivo* fluorescent imaging 4 weeks after implantation. (A) All micro-tissue particle and cell injection graft regions detected by *ex vivo* imaging are electrically coupled to the host, while no patch grafts were coupled to the host. Fluorescence signal of a GCaMP3 graft region vs. time is synchronized with the host electrocardiogram (ECG) for cell grafts at spontaneous rate (B; note that coupling persists during premature beats) and with 6 Hz stimulation (C) and for micro-tissue particle grafts at spontaneous rate (D) and 6 Hz stimulation (E). Epicardial patch grafts are easily detected during imaging but are uncoupled at a slower spontaneous rate (F).

**Fig 6:**
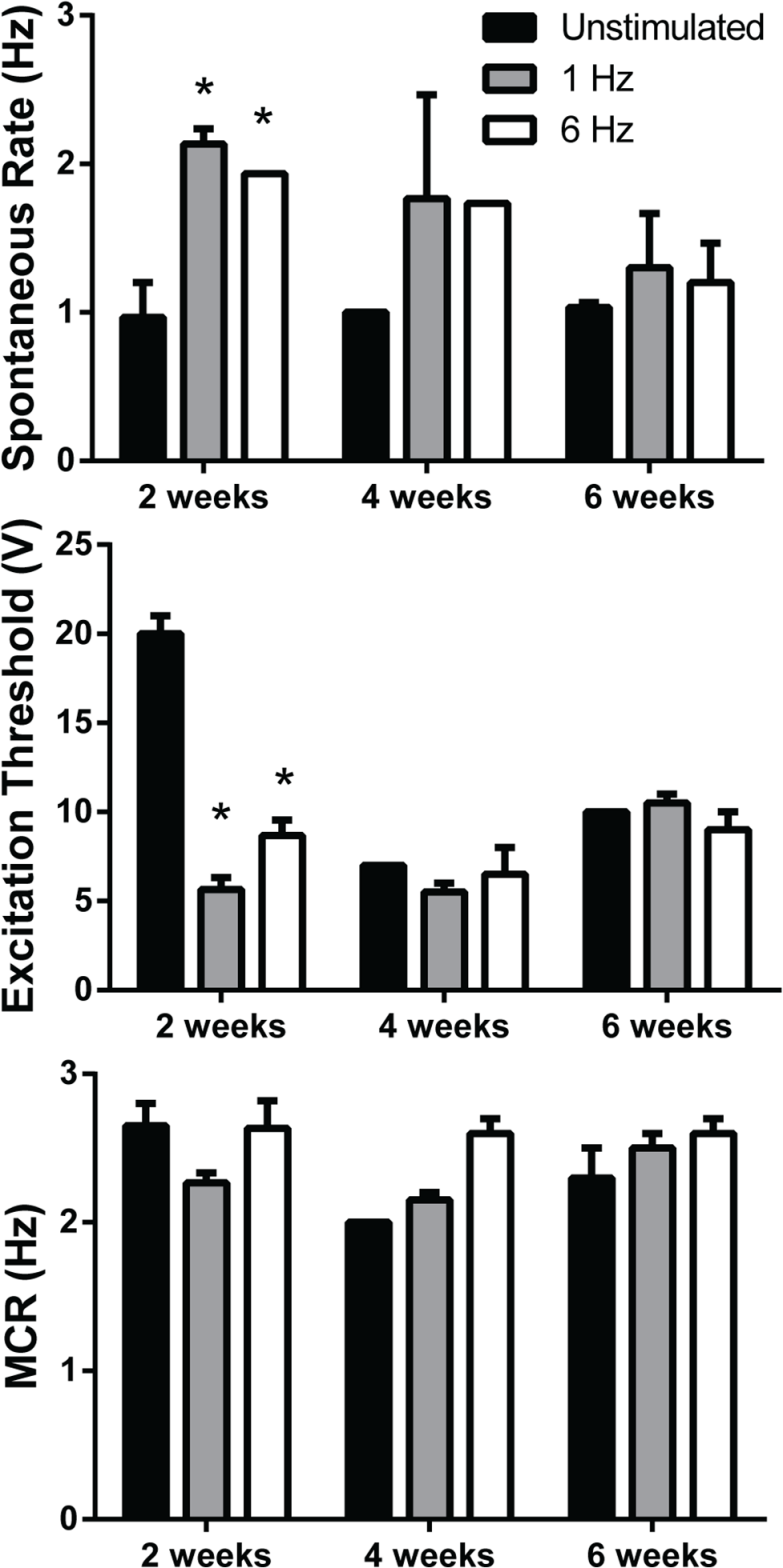
Excitation response of stimulated hESC-cardiomyocytes in 2D culture *in vitro*. Spontaneous contraction rate was higher at 2 weeks with pacing at 1 and 6 Hz (*P < 0.05). Similarly, excitation threshold (ET) was significantly lower after 2 weeks of 1 and 6 Hz electrical pacing compared to unstimulated control. However, maximum capture rate (MCR) was not different among groups at any time point and all significant differences are lost after 4 weeks in culture.

Prior to cardiomyocyte grafts producing mechanical support to improve global heart function, electrical coupling between engrafted cells and host tissue is required. We examined the electrical coupling of GCaMP3 hESC-cardiomyocytes in all three implantation groups using *ex vivo* imaging of the GCaMP3 fluorescent signal in Langendorff-perfused hearts. It was most difficult to detect the grafts in the dispersed cell injection group, where only 4 of 8 hearts had grafts detected from the epicardial surface, although 7 of 8 hearts had grafts detectable by immunohistochemistry (Fig 5A). For both micro-tissue particles and epicardial patches, 6 of 8 hearts had grafts detectable by GCaMP3 imaging, while 7 of 8 hearts had grafts detectable by immunohistochemistry. The *ex vivo* imaging results and maximum capture rates are reported in Table 1. All detectable grafts in both the dispersed cell and micro-tissue particle groups produced fluorescent GCaMP3 signals that were coupled 1:1 with the heart’s ECG recording under spontaneous rhythm, indicating electrical coupling of the cardiomyocyte grafts to the host heart (Fig 5B–5D, S4 Video and S5 Video). Some hearts exhibited up to 4 detectable graft regions, all of which were coupled with the ECG under spontaneous rhythm. Furthermore, electrical stimulation through the host tissue indicated that grafts could be electrically paced, some up to a maximum of 6.5 Hz. Maximum capture rate did not differ between cell and MTP groups (P = 0.07; Table 1). Some of these grafts were found to be in the scar region, as marked by topical paint on the heart’s surface after imaging (Fig 2E, arrow head). In stark contrast, cardiac patches were not coupled to the host in any of the 6 detected patches and showed spontaneous excitation rates of 68.4 ± 11.5 beats per minute (Fig 5F, S6 Video). One patch had sub-regions of distinct GCaMP3 fluorescence activation (likely due to fragmentation during or after implantation), and none of these were coupled to the host myocardium. We tested the ability of hESC-cardiomyocytes to respond to 1 vs. 6 Hz field stimulation *in vitro* in 2D culture and found reliable capture at 1 Hz but no capture at 6 Hz, even with 6 weeks of stimulation time (Fig 6). Excitation threshold was significantly lower in stimulated conditions relative to unstimulated controls, but these differences were lost after long-term culture and there were no differences in maximum capture rate.

**Fig 7:**
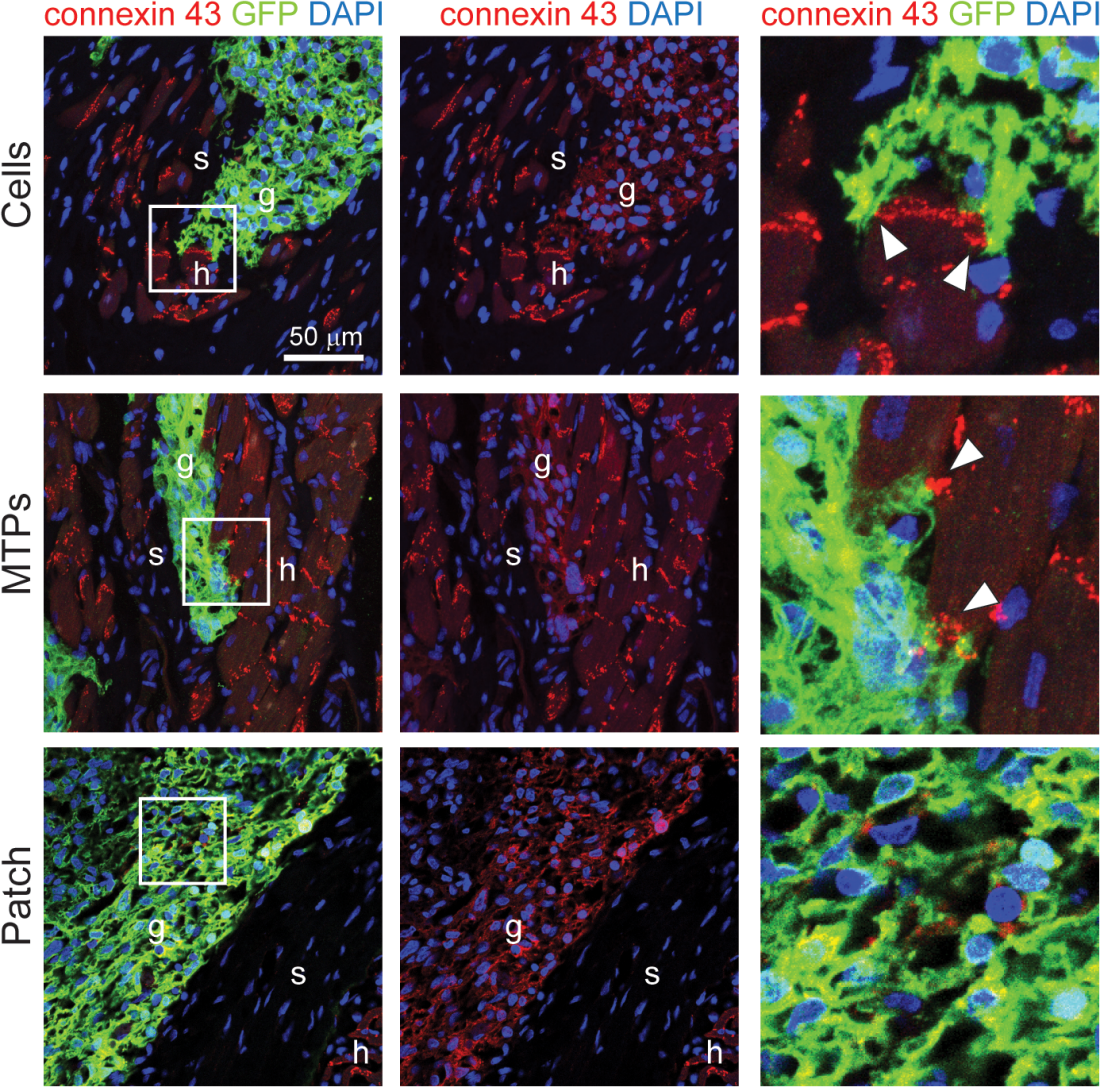
Intramyocardial implants have connexin 43-positive junctions with host cardiomyocytes. Evidence of connexin 43-positive gap junction formation between host cardiomyocytes and hESC-cardiomyocyte grafts was found for cell grafts (A) and micro-tissue particle grafts (B). Boxed region in left column is shown at 4-fold magnification in the right column, highlighting the junctions between graft and host (white arrow heads). Patch implants showed connexin 43-positive regions within the patch (boxed region and right column) and no evidence of gap junction formation with the host, as hESC-cardiomyocytes were physically separated from the host myocardium by scar tissue (C). g, graft; s, scar, h, host. Scale bar = 50 μm.

In electrically coupled dispersed cell and micro-tissue particle grafts, connexin 43 was identified at the border of the GFP-positive grafts between the graft cells and host cells (Fig 7A and 7B), suggesting that physical contact and the formation of gap junctions are required for coupling to occur. Despite the limited sites of connexin 43 between graft and host, this appears to be sufficient for electrical integration. Lower levels of connexin 43 staining are apparent in the graft than in the surrounding host myocardium, possibly due to a lack of structural organization or development of intercalated discs, however this appears sufficient to maintain uniform conduction across the graft (see S1–S6 Videos). In contrast to intramyocardial grafts, there was no evidence of gap junctions between the host myocardium and the epicardial patch implants (Fig 7C). Instead, the patches were separated from the host myocardium by a band of picrosirius red-positive scar tissue (black arrow head, Fig 3A).

## Discussion

Improvement of cardiac function after myocardial infarction will require remuscularization by cell-based therapies that electrically and mechanically integrate with damaged host tissue. The experiments presented here are the first to our knowledge to directly demonstrate electromechanical integration of hESC-cardiomyocytes with rat myocardium after an ischemia/reperfusion injury. This finding supports the use of the rat model for studying human cardiomyocytes in the context of remuscularization and electromechanical integration in the injured heart.

Until now, it was unknown whether human cardiomyocytes (basal rate 60–100 beats per minute) could electrically couple to the rat heart (350–400 beats per minute), which is a widely-used small animal pre-clinical model. We demonstrated here that hESC-cardiomyocytes consistently couple with the rat heart under spontaneous rhythm and can be paced through electrical stimulation of the myocardium as high as 5–6.5 Hz (Fig 5, Table 1). Electrical coupling of hESC-cardiomyocytes was recently demonstrated in acutely and chronically injured guinea pig hearts [6, 7] and in acutely infarcted monkey hearts [8] at lower heart rates. In addition, hiPSC-derived cardiomyocytes have been shown to couple to neonatal rat cardiomyocytes *in vitro*, but spontaneous beating rates were less than 1 Hz [25]. Recent studies have shown the ability to pace hESC-cardiomyocytes up to 6 Hz *in vitro* in 3D engineered tissue using a paced ramp-up from 1 Hz [26], but our attempts to electrically field pace monolayer-plated hESC-cardiomyocytes *in vitro* immediately at 6 Hz (designed to mimic the *in vivo* situation without a ramp-up period) were unsuccessful (Fig 6). There are a number of potential explanations as to why our hESC-cardiomyocytes could not capture the 6 Hz pace *in vitro*, including the 2D environment of our cells, the absence of a ramp-up period, the more terminally-differentiated state of our hESC-cardiomyocytes when experiments are started (day 21–24 of differentiation), or the possibility that the *in vivo* environment provides additional cues not recapitulated *in vitro* in our hands. Preliminary studies by our group demonstrated that at 2 weeks post-implantation, no grafts were coupled by GCaMP3 imaging at this early time point regardless of delivery method (n = 12, data not shown). This raises the possibility that with additional time *in vivo*, the epicardial patches could electrically couple to the host.

Surprisingly, all intramyocardial grafts that were detected by GCaMP3 imaging at 4 weeks were coupled to the rat host heart at the spontaneous heart rate. This finding differs from hESC-cardiomyocyte grafts in the guinea pig heart where 40% of grafts were not coupled after 4 weeks. This difference in ability for grafts to couple to the host in the rat at 4 weeks may be due to the injury model used, which was a cryoinjury model in the guinea pigs and an ischemia/reperfusion injury model in the rat [6], or the distribution of graft within the injured heart. Analysis of the distribution of the intramyocardial grafts in the rat heart shows that at least 20% are located within the scar region (Fig 2F) and some of these were detected by *ex vivo* imaging (Fig 2E, arrowhead). Electrical stimulation of intramyocardial grafts through the host myocardium up to maximal rates of 6.5 Hz demonstrates that hESC-cardiomyocytes are highly plastic and can adapt to their local excitation environment *in vivo*. How this occurs remains to be fully understood. Fatal arrhythmias did not occur in any rats in this study receiving any of the three transplant groups, likely due to the natural resistance to ventricular arrhythmias found in the rodent heart [27]. However, the physiological consequences of these three delivery modes on myocardial contractility remain to be fully described in a comprehensive functional study.

In the current study, we demonstrate that all three delivery strategies for engrafting hESC-cardiomyocytes produce viable grafts, but that only intramyocardially-delivered cells or micro-tissues can be paced through the host rat heart after four weeks. While detection of micro-tissue particle grafts was higher than cell grafts during GCaMP imaging (75% versus 50% detected, respectively), all detected intramyocardial grafts were coupled to the host (Fig 5A). Epicardial patches were not coupled to the host (Fig 5F), and histological analysis shows that all patches have a physical barrier between the engrafted patch and the host myocardium (Figs 2A, 3A, 4, and 7). The absence of coupling with our epicardial patches seems to contrast with the results of Zimmermann et al [17], where engineered heart tissue derived from neonatal rat cardiomyocytes was implanted onto the epicardial surface of infarcted syngeneic rats. They reported that the engineered heart tissues enhanced global systolic function, and they used a multi-electrode array over the epicardial surface to demonstrate that the implanted tissues enhanced cardiac activation patterns and conduction velocities, consistent with electrical integration. However, direct evidence of graft excitation was not used in their study. Our GCaMP3 system is graft-autonomous, as only the hESC-cardiomyocytes express the GCaMP3 transgene. In contrast, the multi-electrode array measures local tissue electrical properties, irrespective of source. Therefore, additional studies will be required to promote electrical integration of epicardially-implanted engineered tissues, such as through direct contact with healthy myocardium or modulation of gap junction formation [28, 29] or scar remodeling [30], while assessing graft-autonomous excitation.

Our study is unique in its approach to compare three different implantation techniques for introducing hESC-cardiomyocytes into the injured heart, and the results bring insights to our cell transplantation approaches for cardiac regeneration. First, the most well-studied and well-established technique of delivering dispersed single cells via minimally-invasive needle injection into the ventricular wall is verified as a viable therapeutic approach. Its simplicity is valuable for clinical translation, and this method has been used in larger animal models and will likely be the first mode of delivery used in human clinical trials. Second, the micro-tissue particles, which were designed to be injectable engineered tissues, were more easily detected by *ex vivo* imaging (compared to dispersed cell grafts) and provide the same minimally-invasive delivery route as dispersed cells. However, we were surprised to find that graft size was not different between micro-tissue particles and dispersed cells by histology, given that cells in micro-tissue particles were not enzymatically dispersed just prior to implantation, which has been suggested to hinder survival upon transplantation [31]. In a study using scaffold-free cardiac cell sheets implanted on infarcted rat hearts, graft size was larger versus injected dispersed cells by *in vivo* bioluminescence imaging [32], suggesting that tissue engineering can produce larger grafts depending on tissue assembly and implant approach. However, electrical integration was not investigated in that study even though whole heart function as measured by echocardiography did not decline with cell sheet implant [32], indicating that paracrine effects on remodeling is possible and that electromechanical integration must be assessed in addition to engraftment size and location. Our graft size results suggest that either anoikis in the dispersed cell group was not a major factor in determining engraftment or that cell death equally affected all implant groups. Further, our graft size data suggests that forming cell aggregates prior to implantation provides no additional benefit over implanting dispersed, single cells when pro-survival factors are included. This contrasts a previous study, where functional benefit was observed via echocardiography of aggregated hESC-cardiomyocytes versus injected cells delivered without pro-survival factors, although graft size was not reported and injected hESC-cardiomyocytes showed minimal engraftment [33]. In our study, there is neither benefit nor detriment to implanting cell aggregates versus a single-cell population in terms of graft size and electrical connectivity achieved. Third, cardiac tissue patches provide the most continuous mass of engrafted cardiomyocytes of all delivery strategies tested and generally maintain their physical architecture as previously demonstrated for cell sheets [32] and scaffold-based engineered heart tissue [17]. However, a lack of difference in graft size versus cells or micro-tissue particles suggests that surgical implantation techniques and detection (by methods other than histology) must be used and developed for epicardial patches. Further, the correlation of decreased graft size with increased scar size (Fig 2D) must be overcome, as a desired therapy should result in robust engraftment regardless of infarct size. Future work in cardiac tissue engineering should aim to overcome the negative correlation observed between graft size and scar size. We also emphasize that in this study cell numbers were normalized at the start of tissue engineering procedures, not at the time of implantation into the rat heart, suggesting that the total number of implanted cells could have been reduced in the engineered tissue groups as lower proliferation rates have been previously reported in this context [24]. However, normalizing cell implantation numbers at this time provided more accurate single-cell counts than is possible after tissue formation. Finally, as others have suggested, larger grafts could be obtained if larger cell input numbers were used in any of the groups. In light of this discussion, we believe that cardiac tissue engineering continues to hold promise as the next generation of cell-based therapies. Indeed, macroscopic and microscopic geometry can be controlled in engineered cardiac tissue based on matrix selection and scaffold fabrication [20, 34–37], and multiple groups have demonstrated uniform electrical excitation of engineered human cardiac tissues [16, 38–40]. Implanted engineered tissues are vascularized by the host to some degree as we have previously shown in epicardially implanted patches [19]. Many efforts are underway to develop a more dense and efficiently perfused vasculature within engineered tissues (recently reviewed by Coulombe, Bajpai [41]), and future studies must include analysis of graft perfusion and vascular remodeling, regardless of delivery strategy, to ensure long-term survival and viability of transplanted hPSC-cardiomyocytes.

In conclusion, this study brings insight to the value of different delivery strategies for transplanting hPSC-cardiomyocytes into the heart for cardiac regeneration. We developed micro-tissue particles as a novel strategy to deliver engineered tissue via intramyocardial injection and demonstrate electrical coupling of all intramyocardial hESC-cardiomyocyte grafts to the rat host with maximal pacing rates of up to 6.5 Hz. This study supports the use of the rat as a valuable small animal model to study the electromechanical integration and contributions of grafts to global heart function of all grafts including epicardially implanted engineered tissues. Larger animal models will be required for future FDA approval of cell-based cardiac therapeutics and to assess arrhythmic risk of such therapies, as rodents are less sensitive models of arrhythmic risk (demonstrated by the occurrence of arrhythmias detected in non-human primates but not in mice, rats or guinea pigs [8]). Ongoing studies in the field are required to develop remuscularization therapy to restore contractile function of the injured heart, and the pursuit of electromechanical integration of epicardially implanted engineered cardiac tissue continues to be a promising approach. As we demonstrate in this study, the use of the rat ischemia/reperfusion model is sufficient for these types of studies due to the ability of hESC-cardiomyocytes to electrically couple to the host heart.

## Supporting Information

S1 Video RUES2 hESC-cardiomyocytes expressing GCaMP3 contract *in vitro* and exhibit robust GCaMP3 fluorescence. The video shows a representative culture of contracting GCaMP3 RUES2-cardimyocytes *in* vitro in 2D monolayer culture at low magnification (4x objective). Cardiomyocytes are first shown in bright-field where a wave of contraction can be seen propagating across the field of view, and then the imaging is switched to show the green fluorescent signal. GCaMP3 fluorescence is increased with each calcium transient.
(MP4)

S2 Video Micro-tissue particles contract and show robust GCaMP3 fluorescence *in vitro*. The video shows micro-tissue particles formed in PDMS microwell molds at low (4x objective) and higher (10x objective) magnification. A bright-field view of micro-tissue particle contraction is shown first, followed by the GCaMP3 fluorescence signal. Each microwell is 400 μm x 400 μm.
(MP4)

S3 Video A macroscale cardiac patch contracts and shows robust GCaMP3 fluorescence *in vitro*. The video shows the edge of a cardiac patch at low magnification (4x objective) in bright-field view followed by the GCaMP3 fluorescence signal.
(MP4)

S4 Video *Ex vivo* fluorescent imaging of dispersed-cell hESC-cardiomyocyte graft. Four weeks after implantation, the rat heart was harvested and perfused with buffer on a Langendorff apparatus to perform *ex vivo* imaging. The video shows a GCaMP3-positive graft region that is 1:1 correlated to the host ECG at spontaneous rate.
(AVI)

S5 Video *Ex vivo* fluorescent imaging of micro-tissue particle graft. Similar to S4 Video, this video shows a micro-tissue particle graft identified during *ex vivo* imaging. This graft is 1:1 correlated to the host ECG at spontaneous rate.
(AVI)

S6 Video *Ex vivo* fluorescent imaging of epicardial patch graft. Similar to S4 & S5 Videos, this video shows an epicardial patch graft identified during *ex vivo* imaging. This graft exhibits a robust GCaMP3 signal but is not electrically coupled to the host ECG and beats at a slower intrinsic rate.
(AVI)
